# Adaptation of High-Throughput Screening in Drug Discovery—Toxicological Screening Tests

**DOI:** 10.3390/ijms13010427

**Published:** 2011-12-29

**Authors:** Paweł Szymański, Magdalena Markowicz, Elżbieta Mikiciuk-Olasik

**Affiliations:** Department of Pharmaceutical Chemistry and Drug Analysis, Medical University of Lodz, Muszyńskiego 1, Lodz 90-151, Poland; E-Mails: pawel.szymanski@umed.lodz.pl (P.S.); elzbieta.mikiciuk-olasik@umed.lodz.pl (E.M.-O.)

**Keywords:** High-throughput screening (HTS), cellular microarrays, drug development, toxicity

## Abstract

High-throughput screening (HTS) is one of the newest techniques used in drug design and may be applied in biological and chemical sciences. This method, due to utilization of robots, detectors and software that regulate the whole process, enables a series of analyses of chemical compounds to be conducted in a short time and the affinity of biological structures which is often related to toxicity to be defined. Since 2008 we have implemented the automation of this technique and as a consequence, the possibility to examine 100,000 compounds per day. The HTS method is more frequently utilized in conjunction with analytical techniques such as NMR or coupled methods e.g., LC-MS/MS. Series of studies enable the establishment of the rate of affinity for targets or the level of toxicity. Moreover, researches are conducted concerning conjugation of nanoparticles with drugs and the determination of the toxicity of such structures. For these purposes there are frequently used cell lines. Due to the miniaturization of all systems, it is possible to examine the compound’s toxicity having only 1–3 mg of this compound. Determination of cytotoxicity in this way leads to a significant decrease in the expenditure and to a reduction in the length of the study.

## 1. Introduction

High-Throughput Screening (HTS) is an approach to drug discovery that has gained widespread popularity over the last two decades and has become a standard method for drug discovery in the pharmaceutical industry. It is basically a process of screening and assaying a large number of biological modulators and effectors against selected and specific targets. It is used not only among industrial scientists but also among academic researchers. HTS assays are used for screening of different types of libraries, including combinatorial chemistry, genomics, protein, and peptide libraries. The main goal of the HTS technique is to accelerate drug discovery by screening large compound libraries at a rate that may exceed a few thousand compounds per day or per week. It is of vital importance, because parallel and combinatorial chemical synthesis generates a vast number of novel compounds. High-throughput screening methods are also used to characterize metabolic, pharmacokinetic and toxicological data about new drugs. HTS technology can reduce the costs of drug development [1–6]. HTS consist of several steps such as target identification, reagent preparation, compound management, assay development and high-throughput library screening [2].

The effective nature of HTS for identification of target specific compounds is attributed to its precise focus on single mechanism. The development of this technology is closely connected to the changes in strategy of chemical synthesis. The vast number of compounds produced by combinatorial chemistry and the possibility of testing many compounds in a short period of time by HTS has attracted the attention of many scientists. Various techniques like fluorescence resonance energy transfer (FRET) and homogeneous time resolved fluorescence (HTRF) are available for identification of compounds [2].

At present, high-density arrays of microreaction wells are gaining popularity in pharmaceutical analysis and drug discovery. Initially there were used 96-well plates but now this format of microplates is currently being replaced by higher density microplates with up to 1586-wells per plate. The typical working volume for these microplates is in the range of about 2.5 to 10 μL total volume, a standard volume is 5 μL per well [7]. However, there are still ongoing trends towards miniaturization of plates. Several examples of biological assays in 3456-well microplates have been reported where the total assay volume was 1–2 μL. However, the use of these ultra-high density plates seems to have some technical hurdles [8].

It is possible to screen up to 10,000 compounds per day by means of typical HTS. Ultra High-Throughput Screening (UHTS) can conduct even 100,000 assays per day [1–3].

At first compounds are tested in primary screens which are less quantitative than biological assays. If an examined compound gives a positive result or “HIT” in such test a more precise secondary screening is conducted and calculations of IC50 values are performed. Secondary screening is performed by means of adopted biological and biochemical tests. Assays are mainly of two types either heterogeneous consisting of five steps such as filtration, centrifugation, fluid addition, incubation and reading or homogeneous (true homogeneous) which is simpler and cheaper than heterogeneous. However, heterogeneous assays appear to be more sensitive [1,2].

HTS are frequently performed by means of miniaturized cell-based assays. Cell-based assays enable chemical libraries to be screened for molecules that present different biological activities. Cellular microarrays are used in the pharmaceutical industry and utilize 96- or 384-well microtiter plates with 2D cell monolayer cultures [9,10]. Cellular microarrays comprise a solid support wherein small volumes of different biomolecules and cells can be displayed, allowing the multiplexed interrogation of living cells and, afterwards, the analysis of cellular responses [11]. Different molecules such as small molecules, polymers, and antibodies can be arrayed using robotic spotting technology or soft lithography [12–14]. The strategy of choice is related to the type of application and problem under study, e.g., surfaces on which cells interact are important for maintaining cellular functions, and the features of these surfaces often influence cellular behavior [15,16]. Soft lithography, which utilizes elastomeric materials, has been used to generate micro-bioreactor arrays for HT experiments using human embryonic stem cells [17] and patterned surfaces for the growth of neural stem cells [18].

These tests are also used for the assessment of a compound’s toxicity. For example, Lee and co-workers implemented HT systems that present the effects of human liver metabolism and at the same time enable cytotoxicity of small molecules to be evaluated [19,20]. Cellular microarrays are also utilized in small molecule screening [21]. One example of such utilization might be a system for screening of small molecules in mammalian cells [22].

Reagents in HTS assays such as enzymes (e.g., tyrosine kinase) cannot be contaminated and have to be optimized. For this purpose Aptamers (nucleic acids) are used due to the speed of their identification, their high affinity for protein targets, and their compatibility with various detection strategies [1].

Currently we may be seeing a trend towards automation and miniaturization of HTS techniques [3]. Miniaturization of bioanalytical processes has become an important area in research with particular focus on laboratory-on-a-chip technology [23]. Advantages of these analytical systems include a reduction in manufacturing costs, ease of transport, and minimal space requirements in the laboratory. Furthermore, miniaturization enables the desired screening rates to be obtained, but on the other hand, it may contribute to long design and implementation time, non-stable robotic operation, and limited error recovery abilities. In the process of automation there are involved multiple layered computers, various operating systems, a single central robot and complex scheduling software. A central robot is equipped with a gripper that can pick and place microplates around a platform. The duration of a single run depends on the type of assay, and during it there are processed from 400 to 1000 microplates. At the beginning of the experiment the screener loads the robotic platform with microplates and reagents and afterwards the assay is processed. Microplates are then passed down a line in serial fashion to consecutive processing modules. Each module, equipped with its own simple pick and place robotic arm and microplate processing device, provides one step of the assay [1]. In this article we present utilization of HTS assays in the assessment of toxicity and drug development. We describe the application of HTS in the determination of modulators of drug-metabolizing enzymes, and in the evaluation of genotoxicity. We also display HTS assays for channel and receptor targets. The next part of the manuscript is focused on broad pharmacological profiling, complex cellular toxicity assays and model organisms’ cytotoxicity assays. We also emphasize the role of HTS in drug development.

## 2. HTS in Toxicology

Humans are exposed to many foreign substances which have the potential to be harmful if they penetrate the body’s natural defenses and enter the bloodstream. Substances may enter the human body via three main ways: inhalation, absorption, and ingestion. When inhaled, a substance must pass through the airway, avoid being trapped in mucus, and enter the body through the alveoli in the lungs. A substance can be absorbed through the epidermal layer, first passing through a thick layer of epidermal tissue. The most common way that foreign substances enter the body is ingestion. In order to enter the bloodstream chemicals must be first absorbed through the intestinal walls.

Toxicology is a field of science that examines the influence of exogenous substances on living organisms. Since the 1950s animal tests have been used for the prediction of the toxicological potential of chemical substances. These tests have been developed to evaluate oral, dermal and ocular toxicity. Moreover, they enable the estimation of immunotoxicity, genotoxicity, reproductive and developmental toxicity, and carcinogenicity [24]. Toxicological tests enable the understanding of molecular targets of chemicals’ toxicity, but data obtained in such studies are frequently limited and are characterized by questionable relevance to the human condition. It is not known whether animal data are relevant to humans and whether application of high doses of substances are relevant to lower doses administered to humans. Animal studies also do not include variation in sensitivity among the human population, e.g., between infants, children and adults. Furthermore, toxicity researches, mainly based on vertebrate animals, are very expensive and require a long time. Another limitation of these studies is there is a large number of chemical compounds, commercially used, of which toxicity should be determined [24,25].

Advances in stem cell biology introduce new opportunities for toxicity testing of chemical compounds. It is extremely important in the field of drug discovery to obtain information concerning potential human safety risks, before heavy commitments are made to investment in particular compounds. Human stem cell (hESC and iPSC)-derived models are being evaluated for their potential to predict human organ-specific toxicities. The model cell lines need to be produced in ways compatible with industrial high through-put screening formats. Fundamental understanding of differentiation programming applied to organ-specific cells of interest is a continuing challenge. Furthermore, there should be established biological and functional differences between cells derived from embryonic or mature cells originating via induced pluripotency [25].

Over the past two decades scientific efforts have been made to develop innovative methods for screening compounds against a large number of potential therapeutic agents. Advances in molecular biology, bioinformatics, and systems biology have led to the application of new methods in the field of toxicology. *In silico* toxicology methods such as computational toxicology, predictive quantitative structure-activity relationship (QSAR) modeling of toxicity and predictive ADME-Tox are currently used in the pharmaceutical industry at the design stage to establish lead compounds with low toxicological potential. *In silico* methods are one of the few techniques that have the potential to significantly improve drug discovery and development. Furthermore, these methods enable the prediction of toxicity from chemical structure. They contribute to the early identification of serious toxicological issues before significant investment of time and financial resources are spent in clinical trials. The advantages of these methods are low costs, standardization, minimal equipment needs, and short time of execution [26–29]. Table 1 presents available *in silico* systems for toxicity predictions.

Another method is HTS which enables the estimation of the potential for toxicity and the understanding of mechanisms of action of a large number of chemicals [24,47–49].

HTS techniques are used in the pharmaceutical industry for screening of enormous numbers of compounds in the drug development process. Chemicals are chosen to cover large areas of chemical diversity and to broadly probe biological function without earlier assumptions. These compounds are tested against different biological targets usually in 96- and 384-well plates, but 1536- or 3456-well plates are also used. Detection systems in HTS are frequently fluorescence, scintillation proximity assays (SPA) and luminescence. These methods facilitate simple and convenient assay procedures and provide high levels of sensitivity. The whole process relies on automation and robotics, thus there is a possibility to test from thousands to a million samples per day. This is possible due to the use of a wide variety of HTS bioassay screens which measure biochemical activity and different cell functions. Generally, HTS assays can measure direct binding to key targets, changes of specific biomarkers, or cellular alteration such as cell shape changes or cell death.

These assays enable structure-activity information to be obtained. The next step of analysis is matching these chemical-activity profiles with proper reference toxicological data. Establishment of a chemical structure and biological activity interface results in a comprehensive insight into activity mechanisms similar to an integrated animal response. One of the most crucial advantages of HTS screening tests is a substantial reduction in costs and animal use. Furthermore, they allow the examination of chemicals at relevant exposure levels [48–50]. In Table 2 types of screening modes are presented [35].

Nowadays, due to growing societal and ethical concerns, scientists (toxicologists, chemists, and modelers) make efforts to acquire new bioassay profiling data of potential relevance to toxicology. All these novel methods prove to have a significant impact on toxicology; they contribute to significant progress in dealing with the toxicity potential of new compounds. Scientists are now looking for new biomarkers for toxicity that will contribute to high-throughput preclinical safety assessment.

High throughput assays can be simply divided into two categories: functional or nonfunctional. Functional assays, which are more reliable, measure the compound’s activity in modifying the function of a target protein, such as ion currents through potassium ion channels. Nonfunctional tests are mainly used for measuring whether a tested compound binds to the target protein. Examples of nonfunctional assays include binding assays and the measure of fluorescence activity associated with calcium signaling. Below the main types of HTS assays are described.

### 2.1. HTS for Modulators of Drug-Metabolizing Enzymes

Drug-drug interactions (e.g., by induction or inhibition of certain enzymes) may contribute to disruption of the normal kinetics of drugs which may result in increased risk of occurrence of adverse effects and as a result affect the clinical development of novel therapeutics. Thus, HTS approaches have been developed in order to determine the activity of chemicals towards modulating the drug metabolizing enzymes. HTS assays which utilize the activity of cytochrome CYP enable the establishment of pharmacokinetic drug interaction and are of vital importance in the drug development industry.

Low-throughput methods combined with liquid chromatography/mass spectrometric methods (LC/MS/MS) allow the determination of the effect of CYP enzymes on examined molecules [51,52]. In these assays hepatocytes are frequently used which enable the acquirement of information concerning II phase of a drug’s metabolism. It is possible to compare in one assay the cytotoxicity of chemicals to the cell line with and without CYP enzymes [53]. Apart from the LC/MS/MS method, there is a possibility to measure the CYP-mediated metabolism by means of fluorescent method. Such assays can be miniaturized using low reaction volumes which enable screening in 1536-well plates [54,55].

There are also available tests which couple measurement of cytotoxicity endpoints with CYP catalysis (e.g., the MetaChip system) [56]. The IdMOC system, a co-culture of five cell types and hepatocytes, enables the evaluation of the effect of metabolites of studied chemicals on a variety of cells which represent different tissues [57].

HTS luciferase reporter gene assays performed in liver cell lines, are used to identify activators of PXR (pregnane X receptor), a nuclear receptor, which contributes to induction of mRNA for a variety of CYPs. There have been reported strong correlations between inducers of the PXR reporter gene activity and the ability to induce CYP3A. For confirmation lower throughput enzymatic activity assays are used [58,59].

Nowadays we are observing an increasing demand for the understanding of individual genetic variability in the development of various diseases and in drug response and toxicities. Thus, screening of different genetic polymorphisms in large populations is a major goal that would facilitate this process. The increasing interest in these pathogenetic studies has resulted in greater demand for broad genome association studies and as a consequence led to the development of new and robust high throughput screening methods for genotype analysis. One of these methods is matrix-assisted laser-desorption ionization time-of-flight mass spectrometry (MALDI-TOF MS), which is a powerful technique for DNA analysis. MALDI-TOF MS approaches have been developed for rapid screening of single nucleotide polymorphisms (SNPs), epigenotype analysis, quantitative allele studies, and for the discovery of new genetic polymorphisms. These methods are based on single base primer extension and minisequencing implemented with new chemicals using MALDI-TOF MS and include photochemical and other chemical and enzyme cleavage strategies that facilitate sample automation and MS analysis for real-time genotyping and resequencing screening [60].

Cytochrome P450 (CYP) enzyme system can be encoded by the P450 gene family. It is one of the widely studied topics in drug development. Several of the drugs metabolizing enzymes, which belong to the CYP family, are polymorphic which means that they have more than one variant of the gene. Despite similar functional properties each of the CYP isozymes is different and has a distinct role. Therefore, individual differences in the efficacy of drug treatment, adverse effects and toxic effects may occur. It is also worth emphasizing that some drugs are metabolized by more than one isozyme, which may contribute to metabolism-based drug-drug interactions. Furthermore, some drugs might be inducers or inhibitors of specific isozymes which lead to alteration in metabolism of other drugs—substrates of these isozymes. There is a wide variety in the expression, activity and concentration of specific isozymes among individuals, species and ethnic groups. The expression of these enzymes is altered by species specificity, genetic polymorphism, age, diseases and environmental inducers. A possible example is CYP450 enzymes, variability associated with this enzyme group results in marked differences in response when the same drug is administered to different people. In the case of CYP450 enzymes there are three major groups of metabolizers: extensive metabolizers, poor metabolizers and ultra-sensitive metabolizers. Genetic differences among individuals can lead to an excessive or prolonged therapeutic effect or toxic overdose [61]. In the drug development process it is crucial to have information on the enzymes responsible for the metabolism of a drug candidate at the earliest stage. Generally, genetic polymorphism contributes to high inter-individual variability and potential for drug-drug interactions. Genetic information is used to establish the response of individuals and populations to drugs. Furthermore, metabolite profiles are important for the design of pro-drugs and pharmacologically active metabolites. For *in vitro* studies before pre-clinical screening low-throughput assays are performed. Information obtained by incubating a tested drug with an appropriate system can be used to design safer and more metabolically stable drugs. Currently there is a wide variety of hepatic *in vitro* systems which differ in biological intricacy. To study multiple aspects of drug metabolism cell cultures or cell suspensions are used. Hepatocytes are utilized for studying Phase I and Phase II reactions. For drug metabolism studies primary cell lines are used which are isolated from fresh liver tissue. These systems can be used immediately after isolation or culture for long-term studies. However, cultured cells lose the enzymatic activity rapidly with time. Thus, there is a great need to improve stabilization of P450 activity [61]. Marks *et al.* developed and characterized a fluorescence-based HTS assay employing recombinant human CYP2B6 and 2 novel fluorogenic substrates (the Vivid CYP2B6 Blue and Cyan Sub-strates). Developed assays have been proven to be robust and sensitive, and allow screening in HTS mode of a large panel of compounds for CYP2B6 metabolism and inhibition [62].

### 2.2. Genotoxicity Assays

Genetic toxicology is the scientific discipline the aim of which is to establish the effects of chemical, physical and biological agents on the heredity of living organisms. For measurement of genotoxicity of chemicals the use of the Ames bacterial reverse mutation test, the mouse lymphoma *tk* gene mutation assay (a negative selection for loss of the functional thymidine kinase gene), and the micronucleus clastogenicity assay are employed. The Ames test, the simplest and quickest of the existing genotoxicity assays, is capable of detecting point mutations and frame shift mutations. However, it does not detect chromosomal rearrangements or double strand breaks. In the micronucleus assay double strand breaks contribute to formation of chromosomal fragments that are not attached to microtubules during metaphase, and are not pulled to opposite poles before cell division. These chromosome fragments migrate outside the normal nucleus and can be observed microscopically as micronuclei. This assay is prone to false positive results which occur when an undamaged but lagging chromosome forms a micronucleus and false negative results which are caused because the micronucleus assay detects only double strand breaks [63]. Methods listed above possess several drawbacks, such as high costs, low specificity and sensitivity. Furthermore, these tests do not allow the screening of a large number of compounds [64].

Scientists’ efforts have led to the development of high-throughput genotoxicity assays which allow screening of a greater number of chemicals. One of such tests is the Ames II assay, adaptation of the previous test which has a very good conformity to the standard Ames testing procedure, decreases the amount of test compound required for a study and is compatible with limited automation [65,66].

Replacement of traditional microscopy by automated cellular imaging enabled higher throughput and contributed to a lower amount of compound (approximately 3 mg) in the micronucleus clastogenicity assay [67].

Ritter *et al.* developed an integrated higher throughput method for the comet assay which is a method for determination of DNA damage *in vivo* and *in vitro*. The scientists’ efforts have contributed to a faster and easier slide-production, smaller amount of cells needed, higher amount of comets quantified, and a fully automated analysis of comets. According to the research results the introduced method was characterized by high reproducibility, flexibility, and efficiency. Thus this procedure can be used as an automated analysis method in HT genotoxicity studies *in vitro* [68].

Evan’s team introduced a new assay system in which the chicken DT40 B cell line was used. This cell line has several significant advantages that make it suitable for genotoxicity studies. It was reported that the implemented assay provides enhanced sensitivity by means of genetically defined and phenotypically characterized mutants, which are defective in DNA repair pathways. Moreover, utilization of DNA repair proficient wild-type cells as a negative control, contributes to minimization of false negative outcomes. Evan’s method enables the establishment of mechanisms of genotoxicity and the extrapolation of the results to the human context [63].

The effects of chemical exposure on gene regulation might be measured also by means of reporter gene-based systems. These systems monitor expression of toxicity markers such as those associated with tumorigenesis, cytokine release and transcriptional activation, which relate to carcinogenicity, mutagenicity, inflammation and endocrine disruption. The use of *in vivo* reporter based assays and the development of transgenic animals constitute a reduction and refinement of traditional rodent bioassays. These *in vitro* assays do not only provide relevant toxicological information, but also facilitate the establishment of mechanisms of toxicity. Despite the fact that reporter gene-based systems have been developed, it is clear that there is no ideal *in vitro* or *in vivo* system to reliably assess genotoxicity. Thus, there is an urgent need to focus on the discovery of tissue-specific toxicity biomarkers so that sophisticated human cell line-based assays can be developed, and validated for regulatory toxicity testing [69].

HTS is a testing strategy that enables a broad probe of biological targets, pathways, and mechanisms in relation to toxicity endpoints, including genetic toxicity, for a large number of compounds. Genotoxicity screening, possessing a unique placement in toxicology, might be regarded as a front-line safety assessment tool. Of vital importance is also the relation of genotoxicity mechanisms with carcinogenicity.

### 2.3. Ion Channel Targets and Receptor Targets

Ion channels are a class of membrane-spanning protein pores that mediate the flux of ions in different cell types. There are different types of ion channels throughout the body, and apart from setting the resting membrane potential and controlling cellular excitability, ion channels also mediate critical physiological functions such as heartbeat, signal transduction, cell secretion, and gene expression. Ion channels use a variety of “gating” mechanisms to open and close their pores in response to biological stimuli such as ligand binding or membrane potential changes. Some of these channels have emerged as attractive drug targets and to realize their full potential as a target class, scientists are actively searching for drugs which could target the specific states of ion channels. It would allow the fine-tuning of drug effects as a function of the degree and frequency of channel activity [68,70,71].

The patch-clamp method is the current gold standard technique for probing ion channel activity. This method allows the measurement of small ion currents with millisecond temporal resolution with simultaneous control of the membrane potential. Automated patch clamp (APC) instruments, in comparison with manual patch clamp, have a 10–100-fold improved throughput [72]. On the other hand, the highest throughput using commercially available APC instruments is only 2000 data points per day. Furthermore, these instruments are rather costly. Thus, APC instruments are primarily used for lead optimization, hit confirmation, and safety screening. As a result, developments in APC technologies are focused on higher throughput, lower cost, and miniaturization [73].

In comparison with the patch-clamp method, optical ion channel assays can achieve a throughput of 100,000 analyses per day. Moreover, they are cheap, simple to perform, non-invasive, amendable to miniaturization and can test multiple cells at a time. A major problem for optical assays is the inability to control the membrane potential and interrogate ion channels in different conformational states [74]. Calcium flux assays are important techniques for Ca^2+^-permeable ion channels because of the availability of excellent Ca^2+^-specific indicators and a large dynamic range of intracellular Ca^2+^ concentrations. There are two main approaches to measure intracellular concentration of Ca^2+^: using organic dyes or genetically encoded proteins with either natural or engineered Ca^2+^ sensitivity [74,75].

Potassium ion channels have attracted huge attention of scientists, as targets for therapeutic indications as well as for safety profiling. In contrast to Ca^2+^-permeable ion channels, no equivalent resources are available for potassium channels. In order to facilitate the pharmaceutical development of potassium channel modulators, high throughput potassium-specific optical assays are critical. One of the major obstacles in designing such assays is the shortage of K^+^-specific fluorescent indicators which are capable of detecting narrow physiological variations of extracellular K^+^ concentration [73].

Assays have also been developed which are aimed at ion channels in order to establish whether a tested compound causes cardiac arrhythmias. In use are binding assays, ion flux assays, fluorescence-based assays, and automated patch-clamp instrumentation. For example the receptor-binding assay detects chemicals that bind to the receptor and is sensitive, whereas it may not induce prolongation of a QT section. As a consequence, compounds active in this test must be examined also by voltage-clamping electrophysiology measurements [76].

Another test is the rubidium flux assay. It guarantees limited throughput and a functional endpoint. However, the main problem is its automation because a flame atomic absorption spectrometer as a detector is required [77].

There are also used fluorescent, voltage-sensitive dyes combined with a fluorometric measurement for potassium ion channel hERG which are sensitive, but simultaneously unreliable with regard to patch-clamp values [78].

By means of high-throughput, gene reporter assays utilizing luciferase or β-lactamase can easily screen compounds with both agonist and antagonist activity for these enzymes [79,80].

Tests have been developed based on protein:protein interactions (domains of the nuclear receptor react with the coactivator protein). These interactions can be measured by the following methods: fluorescence polarization with a fluorescently labeled small peptide [81], fluorescence resonance energy transfer using fluorescently labeled receptor and coactivator [82], or Amplified Luminescence Proximity Homogenous Assay [83].

Traditional receptor binding assays utilizing radio-labeled ligands can be configured for high-throughput tests by using scintillation proximity assay beads [84].

To improve and accelerate the development of new ion channel modulators, there is a need for screening technologies that would offer both high throughput and high information content in a cost effective way.

### 2.4. Broad Pharmacological Profiling

Apart from well-defined and characterized molecular targets with established associations towards toxicity, there is a need for broad screening of chemicals against a panel of molecular targets. Extensive *in vitro* pharmacology profiling of new chemical compounds during early phases of drug discovery has become an essential tool to predict a broad spectrum of clinical adverse effects. State-of-the-art assays and rapidly expanding knowledge about different types of receptors, ion channels and enzymes have made it possible to implement a large number of assays addressing possible clinical activities. These assays can potentially avoid late, high attrition rates, making drug development more cost effective. Furthermore, safety pharmacology profiling of compounds enables the acquirement of receptor-, enzyme-, transporter- and channel-related liabilities of compounds. It has been suggested that this approach would be useful for the estimation of the toxicological potential of environmental chemicals [85]. There is available a large number of these tests, e.g., kinases, proteases, nuclear receptors, phosphatases, phosphodiesterases. A panel of 92 ligand-binding assays was used for identification of relationships between molecular structure and biological activity profiles [86].

An example of the importance of broad pharmacological profiling is the case of CGP 71683A, which is a potent and highly selective neuropeptide Y (NPY) Y5 receptor antagonist. In animal experiments this compound was shown to reduce food intake and lower body weight [87,88]. CGP 71683A exhibited very little activity towards Y1,2,4 receptors and reduced NPY-induced feeding. Thus, this tested compound was regarded as a gold-standard reference compound to study the effects of a selective Y5 receptor inhibition on food intake. Chronic treatment with CGP 71683A caused loss of activity and food intake returned to normal which was explained by the effect of the activation of counter regulatory systems. Although this seemed to be reliable, it remained unclear why the body weight remained low. Although Zuana *et al.* confirmed the selectivity of CGP 71683A within the NPY receptor family, in a broad binding assay panel they estimated high affinity to the muscarinic receptor, the serotonin uptake recognition site and to a lesser extent to α2-adrenergic receptors. Furthermore, morphological observations suggested that this compound caused brain inflammation, which could have contributed to the prolonged weight loss in animals [89]. The study performed by Zuana and co-workers highlights the importance of determining the full pharmacological profile of compounds, especially when complex mechanisms contribute to the development of certain biological activity and when experimental data are only interpreted with some difficulties.

Together with other *in vitro* assays focusing on toxicology and bioavailability, broad pharmacological assays provide a powerful tool to aid drug development. Several commercial databases have been created containing HTS data which are extremely helpful to the development of computational models to establish toxicological potential [47].

### 2.5. Complex Cellular Toxicity Assays and Model Organisms’ Cytotoxicity Assays

Cell-based assays have gained an established position in drug screening in the pharmaceutical industry. These assays enable the evaluation of potential drug targets by functionally characterizing their effect in cells and by assessing specificity and efficacy of drug leads. Cell-based assays provide information on the nature of the pharmacological activity of a compound at a specific receptor, ion channel or intracellular target. Cell-based screening approaches give information on the ability of the chemical compounds to penetrate the cell membrane, as well as an acute cytotoxicity profile. Furthermore, these assays allow the identification of the targets for drugs of unknown mechanism of action and performance of ADME-Tox of potential drugs. It is worth emphasizing that the membrane permeability and cytotoxicity data obtained from cell-based assays cannot be considered as definitive indicators of absorption or toxicity properties of lead compounds. However, such information provides an alert for an encumbered chemical series early in the lead generation process.

Cell-based assays contribute to a high level of complexity and possess several advantages over simple biochemical tests. Cellular models may be regarded as more suitable for detecting toxicity because in opposite to *in vivo* environment, there are no homeostatic mechanisms which could provide a buffering capacity against damages caused by xenobiotics. A variety of different cell lines have been used to predict organ specific toxicity. Furthermore, different species of cell lines are relatively easily accessible and this diversity enables comparison of compounds’ activities among various species. Such research might constitute valuable help in eliciting the reliability of the extrapolation of results from animal tests to humans [47].

In the early stages of the use of cellular models utilization of ATP content, membrane leakage, and cell number as cytotoxicity endpoints was made. These studies were appropriate for evaluating acute toxicity, but did not deliver information regarding the mechanisms of toxicity. Recent efforts and new procedures such as a wide array of cell signaling, stress-response pathways, contributed to greater understanding of toxicity mechanisms.

In Table 3 classification of cell-based assays is presented [1].

There are two types of HCS (High Content Screening). The first one uses fixed cells with fluorescent antibodies, ligands, and nucleic acid probes; the second utilizes live cells with multicolor fluorescent indicators and biosensors. Recent examples of HCS include a screen for cell migration inhibitors using automated microscopy, a screen to detect chemical suppression of a genetic mutation in zebra fish and the use of gene-expression signatures to identify compounds that induce differentiation of acute myeloid leukemia cells [1,90].

Nowadays, mainly due to high-content imaging (HCI) it is possible to conduct high-throughput, quantitative analysis of cellular phenotypic assays. HCI is a combination of epifluorescence imaging platforms and robust image analysis algorithms. Giuliano *et al.* stated that all gathered information at the cell level might be regarded as high-content screening. Such information include the following parameters: cell signaling pathways, protein expression levels, cell cycle status, receptor internalization, cytoskeletal integrity, energy metabolism status, nuclear morphology, post-translational protein modifications, cell movement, and cell differentiation [91]. HCI is usually used to evaluate the effect of chemicals on a set of parameters measuring cell homeostasis. Such procedures enable efficient screening of various compounds. Furthermore, simultaneous measurement of different parameters results in increased sensitivity [92].

The National Cancer Institute created a database of tens of thousands of chemicals which showed activity of growth inhibition on 80 tumor cell lines. This database enabled the understanding of the mechanism of toxic activity of studied chemicals which were grouped on the basis of structural information and biological properties by means of self organizing maps (SOM’s) [93]. This technique was used by Glover’s team. They studied a group containing a number of known inhibitors of mitochondrial complex I of the electron transport chain. They selected ten chemicals with unknown mechanism of action and subsequently five of these appeared to be potent inhibitors of complex I activity [94].

This procedure was successfully used also by Berg *et al.* in order to group compounds by mechanism of action. Exploited HTS panel comprised four human cellular assays and enabled the measurement of multiple inflammation related endpoints. Forty four compounds were classified successfully according to mechanisms unrelated to inflammatory system modulation and thus it could be concluded that this approach may contribute to understanding of mechanisms of toxicity [95]. In 2006 MacDonald’s group established the activity of over 100 drugs in 49 different human cell-based assays at several time points. These tests were based on protein (involved in cell-cycle, apoptosis, mitogenesis, proteolysis, GPCR signaling, cytoskeletal function, DNA damage, nuclear receptor signaling, stress and inflammation) interactions. After analysis it was reported that for some of the drugs new activities were discovered [96].

Dynamic monitoring of cytotoxicity by microelectronic sensors might also be used for characterization of the toxicological mechanism. For this purpose 96- or 384-well plates can be used, where cell viability, morphology and adherence are measured. One of the most important features of this procedure is the possibility to use any attached cell type [97].

Due to the fact that even complex cell culture assays cannot reliably model the higher level interactions which are presented in living organisms, HTS approaches have been developed using whole animals in the form of model organisms.

As model organisms invertebrates can be used, e.g., the yeast, Saccharomyces cerevisiae [98], and nematode, Caenorhabditis elegans [99]; and vertebrates, such as the zebrafish, Danio rerio [100]. In Table 4 there is presented the characterization of model organisms used in HT screening.

These model organisms, mainly during developmental stages, may interrogate vertebrate animals and thus contribute to easier access to a wide array of chemicals. By explicating the mechanisms involved in the response of the organisms to chemicals, then evaluating and testing that mechanism across species against any molecular targets, it is possible to establish a risk assessment.

In comparison with studies on larger vertebrates, these studies are inexpensive and capable of testing moderate numbers of chemicals, although not in the traditional HTS range, but are still laborious.

Whole organisms, such as zebrafish embryos, have become attractive model systems for developing disease-related assays for toxicology studies. Limitations in performing these assays are the availability of fast image acquisition systems with sufficient resolution and depth of field and the analysis of the complicated images to provide fast and accurate quantification of the biology of interest in the assay [109].

Nowadays the trend towards miniaturization of assays may be observed. At present most HTS is carried out in 96-well plate format, but utilization of 384-well plate formats is increasing. This plate format has been established as the format of choice for compound storage and screening assays and is used in various types of biochemical and cell-based assays. An 864-well and 1536-well plate format has also been implemented. However, the utilization of such high density formats is limited by numerous obstacles [1]. Benefits of miniaturization include lower volume of reagents required and faster experimental processing, and as a consequence reduction of cost and time [110].

In summary, computational toxicology, SAR prediction models, toxicogenomics, and HTS testing programs significantly alter the current paradigm of toxicity screening. A major challenge for the field of contemporary toxicology is to embrace computational toxicology, structure-based and *in silico* prediction methods, and new assay technologies that are able to efficiently screen thousands of chemicals [111].

## 3. HTS in Drug Discovery

Drug discovery, placed in the field of medicine, pharmacology and biotechnology, is associated with research on drug targets and mechanisms. In the past most drugs have been discovered by identifying the active ingredient from traditional resources (plants, minerals, *etc.*) or by discovery. Drug-discovery is a highly complex, multidisciplinary and time-consuming program, which typically starts with the identification of suitable drug targets (e.g., biomolecules such as receptors, enzymes and ion channels). The next step is target validation in which it is established whether the target is of relevance to the disease under study. Afterwards modulators of the target have to be identified. Such modulators are agonists or antagonists of receptors, activators or inhibitors of enzymes, and openers or blockers of ion channels. Suitable assays are then developed to monitor the target under study. An example is HTS which exposes the target to a large number of chemical compounds. In this phase “lead” compounds are obtained which are characterized by a certain degree of selectivity for the target. These ‘lead’ compounds are then optimized in terms of their potency, selectivity, physicochemical properties, and pharmacokinetic and toxicity properties. The last phases of the drug discovery processes are human trials [1,112]. High throughput methods are in high demand in drug development today. Their main goal is to accelerate drug discovery by screening large libraries (e.g., combinatorial chemistry, genomics, protein, and peptide libraries) often composed of hundreds of thousands of drug candidates. HTS is playing an important role in early stage of drug development, providing qualitative and quantitative characterization of compound libraries and analytical support for preclinical and clinical ADME studies. Thus, HTS facilitates early elimination of unsuitable compounds [1].

Also *in silico* methods play a vital role for drug discovery. There are several steps, like target identification, reagent preparation, assay development and high-throughput library screening that are obligatory for successful HT assay.

Over the past decade, the HTS strategy has been developed by advances in technology such as automation of liquid handling, creation of novel platforms, and development of analytical tools to deal with massive quantities of data. Thus HTS became indispensable in all stages of drug discovery, from target identification to toxicity evaluation. Miniaturization and automation contribute to cut reagent use and analysis times, minimize or eliminate labor-intensive steps, and dramatically reduce assay costs.

One of the examples of utilization of HTS in drug discovery is high-throughput assay that enables screening against enzymatic targets. This test relies on the biocatalytic conversion of a non-fluorogenic substrate to a fluorescent product and presents broad potential to impact various stages of the drug discovery process, including lead identification and optimization, and ADME/Tox assessment. In this method compounds that most effectively inhibit or activate the enzyme target are identified [113] Apart from fluorescent techniques, especially in the case of requirement of structural characterization of the products, other techniques are used, like mass spectrometry (MS). MS is a suitable method for compound characterization because of its selectivity, sensitivity, resolution, and capability of sample identification and structure elucidation. Furthermore, it is capable of easy and selective separation of target molecules from a complicated mixture, without an extensive sample preparation procedure. Flow injection analysis-MS (FIA-MS) with an eight-probe autosampler enables the characterization of combinatorial libraries in a single 96-well plate in 5 min [114]. An automated MALDI-Fourier transform-MS (MALDI-FT-MS) is capable of analyzing 20 samples in one hour [115]. There are also used other analytical techniques such as liquid chromatography/mass spectrometry (LC/MS) and nuclear magnetic resonance (NMR). LC/MS has become the standard technique to monitor the progress of synthetic reactions in real time and verify the identity and purity of compounds. LC/MS can be applied in metabolite identification in HT assays concerning adsorption, distribution, metabolism and elimination of potential drugs.

High-throughput NMR-based screening is a useful tool for structural characterization of protein-ligand interactions, aiding the identification of compounds that bind to specific protein targets [113,116].

Other combination techniques used in structural characterization of compounds are FIA/DI-MS, MALDI-FT-MS, DI-NMR, HPLC-UV/MS, HPLC-NMR, SFC-MS, and ESI-FT-ICR-MS.

Apart from structural analysis, HTS also supports purity determination of screened compounds. HPLC is a technique utilized for the determination of purity and is capable of high throughput status via reduction in cycle times and development of generic analytical methods [1]. HPLC-MS, the most powerful high-throughput purity analysis method, has been used in the analysis of chiral impurities present in diastereomeric peptide drugs [117].

As ELSD is sensitive to the mass of an analyte, it is a more uniform response which is obtained from small-molecule libraries when compared with UV absorbance, because the extinction coefficient of compounds within the library can vary widely [118,119].

Virtual screening is another technology that has an increasing role in drug discovery, especially in the lead identification stage. It is regarded as a complementary approach to HTS, and when coupled with structural biology, enhances the chances of identification of the lead [120]. Virtual high throughput screening (vHTS) applies *in silico* approaches, such as docking and alignment, to large virtual molecular databases to enrich biologically active compounds in order to yield lead structures [5,121]. The vHTS method can be regarded as a way to screen databases or from another point of view, as a simplified simulation of high-throughput screening assays. It is believed that vHTS integrates computer science with biophysics. It is characterized by the flexibility, cost-effectiveness, and speed of computational algorithms and biophysical knowledge on molecular recognition. Such integration may increase hit rates or enrich hit lists from HTS [122]. An example is platform MolMind which combines *in silico* and laboratory methods. In this platform a genetic algorithm is used to lead a robotic synthesis system; furthermore, chemical and biological screening is used to obtain molecules with the desired properties [121].

Use of information created experimentally or *in silico* in iterative screening procedures contributes to optimization of the efficiency of HT drug-design methods [122].

The HTS methods offer an enormous benefit to the drug discovery field and there will certainly be continuous development of these dynamic and very competitive assays.

## 4. Conclusion

The rapid growth in the amount of chemical and biological data using HT methods in drug discovery makes necessary the use of computational technologies, such as databases, in order to store, manage, analyze, and interpret research results. HTS in its basic form has been used for several decades but currently vHTS is gaining greater importance. There are also available other forms of HTS such as *in silico* screening, virtual screening, library screening, computational screening. These methods differ from each other in the aim of the conducted analyses. The methods utilize data libraries of chemical compounds. Thus, there is the possibility to determine different parameters of designed compounds including basic physicochemical parameters, theoretical affinity for an active site, the capacity of the brain-blood barrier penetration and the theoretical definition of a compound’s toxicity. Computer technologies are used in numerous fields of the pharmaceutical industry such as bioinformatics, systems biology, chemoinformatics, drug design, toxicology, pharmacokinetics, and pharmaceutical formulation. Computational analyses facilitate making decisions, contribute to innovations and learn from failure [123].

Chemical compound libraries arise due to collection of data which was acquired of earlier synthesized compounds and from research conducted by means of various techniques including HTS methods. Screening assays utilize these data and data obtained in *in silico* methods. As a consequence, results of such analyses can be loaded with errors and results of analyses with the same aim and procedures might be inconsistent. For these types of analyses different types of software are used, such as Fred (OpenEyeScientific Software, Santa Fe, USA), Glide (Schrödinger, Portland, USA), LigandFit (Accelrys, San Diego, USA), Quick Explore (QXP), FFLD, Eudock, and ICM-DISCO (Docking and Interface Side-Chain Optimization). On line databases for example Adme Works Predictor (Fujitsu) are also used.

Due to vast computational possibilities concerning the number of chemical compounds of whose parameters can be established in a short time, HTS assays are utilized not only in research and development centers but also in pharmaceutical companies. Thus, it is also a basic tool used in drug development. It is estimated that HTS contributes to savings of 130 million dollars and about 0.8 years of work over the development of a new drug [123]. That is why HTS methods are being continuously expanded. It is expected that in the not-too-distant future we will experience further miniaturization and elaboration of more selective markers.

## Figures and Tables

**Table 1:** Examples of available *in silico* systems.

System name	Description	Reference
QSAR	Structural correlation between compounds and biological activities; enables prediction of various endpoints.	[30]
MDL QSAR	Enables establishment of structure-property relationships, generates new compound libraries, used for prediction of mutagenicity, carcinogenicity, skin sensitization and irritancy.	[31]
PreADMET	Calculation of important descriptors and neural network for the construction of prediction system, used for prediction of mutagenicity and carcinogenicity.	[32]
MCASE	Identifies molecular fragments with a high probability of being associated with a biological activity, used for prediction of mutagenicity, carcinogenicity, teratogenicity, irritancy, maximum tolerated dose and biodegradation.	[33,34]
TOPKAT	Employs cross-validated QSTR (quantitative structural toxicology relationship) models for assessing various measures of toxicity, used for prediction of mutagenicity, carcinogenicity, teratogenicity, lethal dose, skin sensitization, and environmental toxicity.	[35]
Lazar	Searches the database for compounds that are similar with respect to a given toxic activity, used for prediction of mutagenicity, carcinogenicity and hepatotoxicity.	[36]
ToxScope	Correlates toxicity information with structural features of chemical libraries, and creates a data mining system, used for prediction of mutagenicity, carcinogenicity, irritancy, and hepatotoxicity.	[37]
COMPACT	Identifies potential carcinogenicity or toxicity mediated by CYP450s (Cytochrome P450), used for prediction of carcinogenicity and P450-mediated toxicities.	[38,39]
OncoLogic	Knowledge-based expert system, used for prediction of carcinogenicity.	[40]
MetaDrug	Toxicogenomics platform, used for prediction of ADME-Tox (absorption, distribution, metabolism, excretion-toxicology) properties.	[41]
CSGeno Tox	Encloses electrotopological state indexes, connectivity indexes and shape indices, used for prediction of mutagenicity	[42]
CADD	Computer-aided drug design by multi-dimensional QSARs (Quantitative structure-activity relationship) applied to toxicity-relevant targets, used for prediction of receptor- and CYP450-mediated toxicities.	[43]
DICAS	Has capability to seek local correlations in datasets with large number of attributes, used for prediction of carcinogenicity.	[44]
DEREK for Windows	Knowledge-based expert system, used for prediction of mutagenicity, carcinogenicity, skin sensitization and irritancy.	[45]
HazardExpert	Used for prediction of mutagenicity, carcinogenicity, skin sensitization, irritancy, immunotoxicity and neurotoxicity.	[46]

**Table 2:** Types of screening modes.

Screening mode	Number of samples tested per day	Examples
Low-throughput screening	1–500	Animal models, assays for CYP-mediated metabolism combined with LC/MS/MS
Medium-throughput screening	500–10,000	Fluorescent cellular microscopic imaging assay, assays for determination of catalytic activities of oxygen-consuming enzymes
High-throughput screening	10,000–100,000	Fluorescent enzymatic inhibition assay, luciferase reporter gene assays
Ultra-highthroughput screening	>100,000	β-lactamase cell reporter assay, assay for quantification of 5-HT_2C_ receptor editing

**Table 3:** Classification of cell-based assays.

Type of assay	Description
Second messenger assays	Monitor signal transduction from activated cell-surface receptors, measure fast, transient fluorescent signals.
Reporter gene assays	Monitor cellular responses at transcription/translation level, indicate the presence or absence of a gene product that reflects changes in a signal transduction pathway.
Cell proliferation assays	Monitor the overall growth or no growth responses of the cell to external stimuli, quick and easy to be employed for automation.
High content screening	Analyses cells using fluorescence based reagents, yields information that will permit more efficient lead optimization before the *in vivo* testing, used for multiparametric measurement of apoptosis, which provides information on parameters such as nuclear size and shape changes, nuclear DNA content, mitochondrial potential, and actin-cytoskeletal rearrangements during drug-induced programmed cell death.

**Table 4:** Model organisms used in High-throughput screening (HTS).

Model organism	Characterization	Application	Reference
Saccharomyces cerevisiae	Ease of growth, sequenced genome, availability of a wide range of genetic mutants	Understanding of physiological and pathophysiological processes in higher level organisms; identification of the ability of an apoptosis-inducing chemical to kill cells through generation of reactive oxygen species by the electron transport chain; measurement of hypersensitivity to chemicals; examining the activity of chemicals with DNA-damaging activity	[101–103]
Caenorhabditis elegans	Ease of growth, well characterized, availability of a large number of mutant strains, suitable for RNAi studies.	Understanding of toxic mechanisms by utilization of hypersensitivity and hyposensitivity to certain compounds.	[104,105]
Danio rerio	Utilization of transparent embryo in observing changes to organ morphology, availability of genetic manipulations.	Utilization in environmental toxicant testing and research concerning drug development; observation of malformations caused by chemicals, studies of neurotoxicity; injection with oligonucleotides contributes to a reverse genetics approach	[106–108]

## Abbreviations

FIA: flow injection analysis
DI: direct injection
MALDI: matrix-assisted laser desorption/ionization
FT: Fourier Tansform
HPLC: high-performance liquid chromatography
SFC: supercritical fluid chromatography
ESI: electrospray ionization
ICR: ion cyclotron resonance
